# Mental health during pregnancy and postpartum in women with a history of bariatric surgery: A scoping review

**DOI:** 10.1007/s00737-026-01727-w

**Published:** 2026-05-19

**Authors:** Seyedeh Samira Mokhlesi, Vidanka Vasilevski, Linda Sweet

**Affiliations:** 1 School of Nursing and Midwifery, Centre for Quality and Patient Safety Research in the Institute for Health Transformation, Deakin University, Burwood, Victoria, Australia; 2 Western Health, St Albans, Victoria, Australia

**Keywords:** Mental health, Bariatric surgery, Pregnancy, Postpartum period

## Abstract

**Purpose:**

With rising bariatric surgery rates, more women of childbearing age are becoming pregnant after bariatric surgery. Bariatric surgery is often associated with psychological diagnoses. While mental health issues are common during pregnancy and postpartum, the effects of bariatric surgery on mental health during these periods is unclear. This review aimed to collate and synthesise available literature regarding the impact of bariatric surgery on mental health during pregnancy and postpartum.

**Methods:**

The review followed the Preferred Reporting Items for Systematic Reviews and Meta-Analyses extension for Scoping Reviews framework. A systematic search was conducted across five databases (MEDLINE, Embase, CINAHL, Maternity and Infant Care, and Global Health). It included peer-reviewed primary research studies and conference abstracts published in English that reported mental health outcomes of women during pregnancy and postpartum with histories of bariatric surgery. There were no time restrictions for including studies. A narrative synthesis following the Popay et al. framework was performed to summarise and interpret the findings.

**Results:**

The available evidence suggest that women who have undergone bariatric surgery may be at increased risk of experiencing depression and anxiety during pregnancy compared to women who have not had bariatric surgery. Factors such as marital status, psychiatric history, and smoking exacerbate these risks. Evidence regarding other mental health disorders and postpartum depression was limited and inconsistent.

**Conclusions:**

Pre-pregnancy bariatric surgery may increase the risk of mental health challenges during the perinatal period. Mental health assessments should be integrated into antenatal and postnatal care for women with bariatric surgery histories.

**Supplementary Information:**

The online version contains supplementary material available at 10.1007/s00737-026-01727-w.

## Highlights

Depression and anxiety are more common in pregnancy after bariatric surgery.

Being single, smoking, or past mental illness raise mental health risks in women with a history of bariatric surgery.

Providing targeted perinatal mental health screening and support for women with bariatric surgery is important.

**Supplementary Information:**

The online version contains supplementary material available at 10.1007/s00737-026-01727-w.

## Introduction

The World Health Organization (WHO) reports that the global rate of obesity has more than doubled between 1990 and 2022 (WHO, 2024). In 2022, approximately 16% of adults aged 18 and older were classified as having obesity (WHO, 2024). Women of reproductive age are experiencing higher rates of overweight and obesity, a trend that is rising steadily (Chowdhury et al. 2018; Nglazi and Ataguba 2022). Obesity is recognised as a significant risk factor for various aspects of women’s reproductive health, including fertility, pregnancy outcomes, and breastfeeding (Ogunwole et al. 2021).

Bariatric surgery is a type of surgery which helps people with obesity to lose weight (Shanti and Patel 2019). Bariatric surgery is an effective treatment option for obesity, providing long-term weight management through various mechanisms (American Society for Metabolic and Bariatric Surgery, 2021). These mechanisms include reducing stomach capacity to restrict food intake, reducing energy and nutrient absorption by altering the duodenum, or a combination of both mechanisms (American Society for Metabolic and Bariatric Surgery, 2021). Unlike other weight loss strategies such as dieting, exercise, behavioural changes, or weight loss medications, bariatric surgery provides a reliable way to achieve and maintain significant weight loss, reducing up to 30% of total body weight (Shanti and Patel 2019). With rising obesity rates, the number of women of childbearing age undergoing bariatric surgery has increased over the past two decades (Rottenstreich et al. 2019; Alsuhibani et al. 2023). Weight loss following bariatric surgery can restore ovulation and enhance fertility, thereby increasing the chances of pregnancy in women of childbearing age who have undergone the surgery (Solaiman et al. 2023; Mokhlesi et al. 2024).

Becoming pregnant too soon after surgery when weight loss is at its highest, has been associated with several risks for both the mother and the fetus (Falcone et al. 2018). Therefore, it is recommended that individuals wait at least 12–24 months after surgery to allow for weight stabilisiation and correction of nutritional deficiencies prior to becoming pregnant (Dolin et al. 2021). Women with a history of bariatric surgery who intend to become pregnant, should undergo preconception counselling to be informed about the potential risks of pregnancy after bariatric surgery, such as nutritional deficiencies, internal hernia, and having a small-for-gestational-age (SGA) infant (Falcone et al. 2018). The risk of developing a mental health disorder is another consideration. Evidence has shown that bariatric surgery is often associated with psychological diagnoses (Troisi 2020). Nutritional deficiencies, which are common after bariatric surgery can directly affect mental health in individuals who have undergone the procedure. For instance, low iron, B12, and copper levels are associated with symptoms such as low mood (Ratcliffe 2021). In women with histories of bariatric surgery who become pregnant, mental health disorders can be linked to nutritional deficiencies exacerbated by the physical demands of pregnancy (Morgan et al. 2025), and the challenges they may face in adapting to the emotional and bodily changes associated with pregnancy (Bąk-Sosnowska and Naworska 2023).

Maintaining optimal maternal mental health during pregnancy is crucial, as these challenges may result in adverse health outcomes for the mothers, infants, and families (Alipour et al. 2018). Mental health issues impact nearly 20% of pregnant women during the prenatal and postpartum periods, which can extend up to a year post birth (Alipour et al. 2018). The most common mental health challenges women face during pregnancy include anxiety, depression, and self-harm (Bedaso et al. 2021). Given that pregnancy and postpartum are considered peak periods for the development of mental health conditions, it is important to understand whether having a history of bariatric surgery compounds this risk. To our knowledge, there is no review investigating the impact of bariatric surgery history on mental health during pregnancy and postpartum. Given the increasing prevalence of bariatric surgery and the importance of mental health on the wellbeing and safety of mothers, infants, and families, the aim of this study was to collate and synthesise available literature regarding the impact of bariatric surgery on mental health during pregnancy and postpartum. The review question was “what is the impact of bariatric surgery on mental health during pregnancy and postpartum?”

## Method

### Study design

A scoping review and narrative synthesis was conducted. Scoping reviews are effective for addressing broad questions and identifying literature gaps to inform future research (Tricco et al. 2016). The review adhered to the Preferred Reporting Items for Systematic Reviews and Meta-Analyses extension for Scoping Reviews (PRISMA-Scr) guidelines for systematic reviews and meta-analyses, ensuring transparency and rigor (Tricco et al. 2018).

### Information sources and search strategy

Five databases were searched, including MEDLINE, Excerpta Medica Database (Embase), Cumulative Index of Nursing & Allied Health Literature (CINAHL), Maternity and Infant Care and Global Health. These databases were selected for their relevance to the research question and their broad coverage of surgical, perinatal, and psychological related literature. The literature search was initially conducted in December 2024 and was updated in June 2025 to include the most recent studies.

The search terms and keywords were: “mental* health” OR “mental* disorder*” OR depression* OR “depressive* disorder*” OR “depression, postpartum” OR anxiety OR “anxiety disorder*” OR “mood disorder*” OR psychology* OR psychiatry* AND “bariatric* surg*” OR bariatric* OR “metabolic surg*” OR gastroplasty OR “body weight management” OR “obesity management” OR “gastric bypass” OR “ Roux-en-Y gastric bypass” OR “gastric band*” OR “sleeve gastrectomy*” OR “stomach stapling” AND “perinatal care” OR “postnatal care” OR “postpartum period” OR “prenatal care” OR “prenatal diagnosis” OR pregnancy OR pre-natal OR post-natal OR post-partum OR pre-natal. Additionally, we reviewed the reference lists of the identified papers and used Google Scholar to locate any additional relevant articles that may have been missed. The complete search strategy used for MEDLINE is available in Additional File 1.

### Study selection

We included primary studies of any design that reported on mental health during pregnancy and postpartum in mothers with a history of bariatric surgery. Secondary studies, including reviews and guidelines, were excluded to avoid overlap of findings. The inclusion criteria were: [1] full-text articles and conference abstracts reporting research on mental health during pregnancy or postpartum in mothers who have had pre-pregnancy bariatric surgery, [2] primary studies with quantitative, qualitative, or mixed methods designs, and [3] publications in English. A time limit was not imposed on this search to ensure all relevant studies were considered. Exclusion criteria included book chapters, editorials, letters to the editor, guidelines, study protocols, non-human studies, review articles, or non-English publications.

Following the systematic search, the results were exported to EndNote, where duplicates were removed. All remaining citations were then imported into Covidence for screening and eligibility assessment. Covidence, a web-based platform designed to facilitate systematic evidence reviews, supports effective collaboration among multiple reviewers throughout the review process (Babineau 2014). Two researchers independently reviewed the titles and abstracts in Covidence, excluding studies that did not meet the inclusion criteria. Full text publications of studies that passed the initial title and abstract screening were sought, and two researchers independently assessed them for inclusion. Any disagreements during the screening phases were resolved through discussion or consultation with a third researcher.

### Data charting

A data extraction form was created using a Microsoft Excel spreadsheet to document the key characteristics of each study, along with relevant information on mental health during pregnancy and postpartum in women who have undergone bariatric surgery prior to pregnancy. SM charted the data and discussed the results with VV and LS to reach final consensus. Data extracted from the eligible articles included the first author’s surname, year of publication, study setting, study aims, study population, type of bariatric surgery, the interval between surgery and pregnancy, study design and methods, mental health screening or diagnostic methods and key findings, including the mental health outcomes assessed in each study.

### Quality appraisal

Critical appraisal is optional in scoping reviews (Levac et al. 2010), however, we conducted it to enhance the interpretability of our findings. The included studies were critically appraised by two researchers using the Joanna Briggs Institute (JBI) Critical Appraisal Checklist specific to each study design. These checklists assess overall methodological quality and include items relevant to risk of bias (JBI, 2020).

. Any conflicts that arose during the appraisal process were resolved through discussion. Each article was assigned a quality score upon completion of the assessment. All articles were included to provide a comprehensive overview, however, the quality appraisal findings were used to help interpret and determine the reliability and validity of the included studies and their subsequent findings (Pollock et al. 2022).

### Synthesis of results

The Popay et al. (2017) narrative synthesis framework was used in this review to effectively summarise and interpret the findings from the included studies. This approach provides a clear and organised presentation of the results, and can be applied to a wide range of review questions (Popay et al. 2017). Initially, the findings from the studies were gathered and summarised to highlight key patterns and observations. Then, these findings were compared across studies to identify similarities, differences, and unique insights. In the final step, the findings were grouped into specific themes for a clearer and more organised presentation of the results (Popay et al. 2017).

## Results

**Fig. 1:**
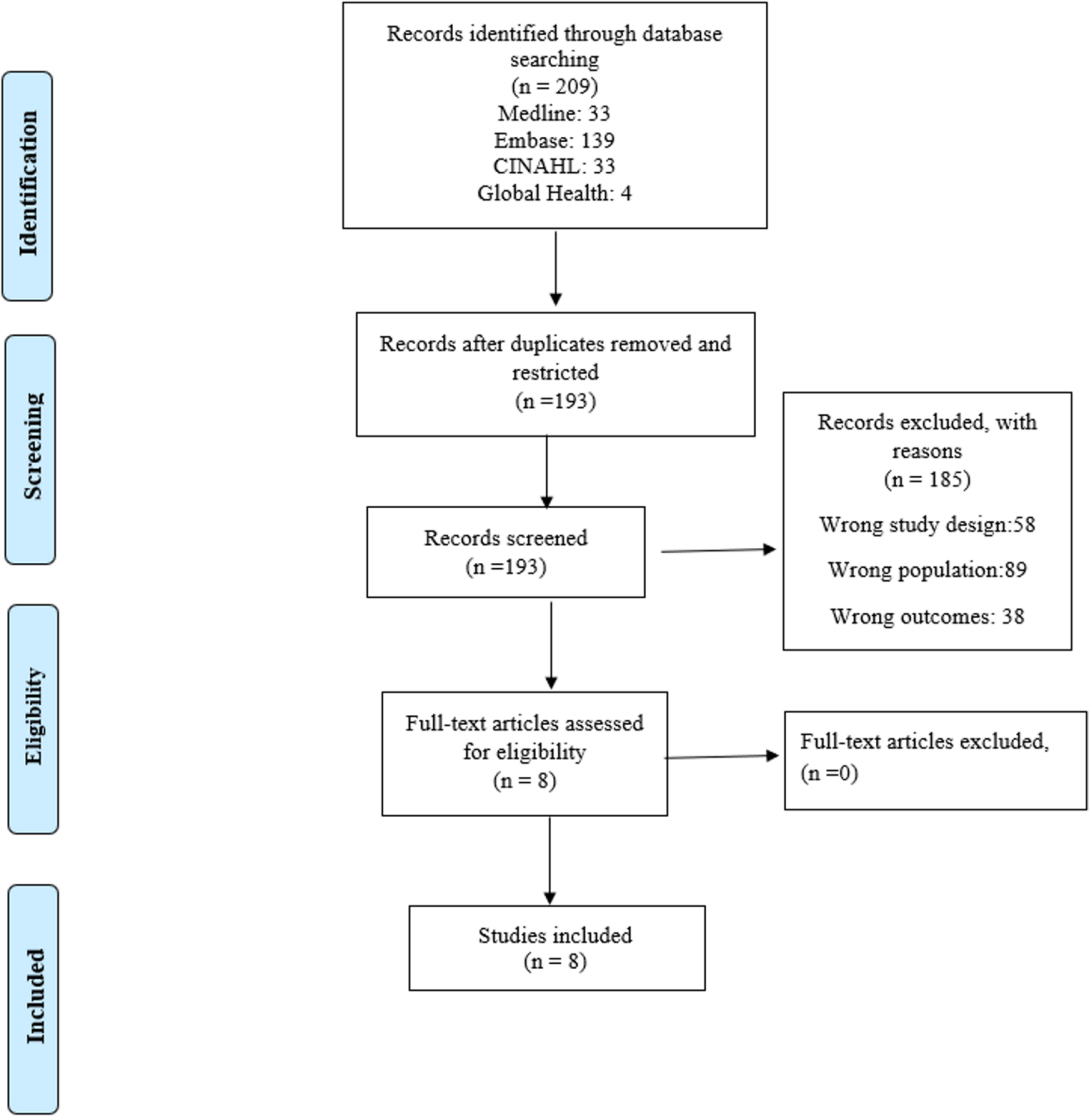
PRISMA literature search and study selection

The initial search resulted in 209 articles, out of which 16 were duplicates. We screened the titles and abstracts of 193 articles and excluded 185 that were irrelevant to our aims. This left us with eight articles for full-text screening. After applying the inclusion criteria, we did not remove any articles, ultimately retaining eight for extraction. The record of the search procedure is illustrated in the PRISMA chart shown in Fig. 1.

The main characteristics of the studies included in the analysis are presented in Table 1. All eight studies were conducted between 2018 and 2022 indicating an increase in research activity in this area in recent years. Most studies were conducted in the United States (*n* = 4, 50%), followed by Brazil (*n* = 3, 37.5%), and Belgium (*n* = 1, 12.5%). Three out of the eight studies (37.5%) were published conference abstracts, with no full text manuscripts found following another search of these authors. The eight included studies comprised four cohort studies, two case-control studies, one cross-sectional study, and one observational investigation. Most studies (*n* = 6, 75%) primarily focused on depression and/or anxiety among pregnant women with a history of bariatric surgery. Two studies reported on the prevalence of other mental health disorders in addition to depression and anxiety, such as eating disorders or alcohol/substance use disorders (Rocha et al. 2022; Yu et al. 2022). Two studies addressed mental health during the postpartum period (Kok et al. 2018; Walter et al. 2021); one of these was a conference abstract (Kok et al. 2018).

**Table 1:** Main information for studies included in the review (*n* = 8)

Authors, Year, Country	Aim	Sample, Demographics	Most common type of bariatric surgery (*n*, %)	The interval between surgery and pregnancy	Design and Method	Screening/Diagnostic Methods	Key Findings
Fatima and Popov (2022), United States	Assessed labour/birth and pregnancy complications in pregnancy following bariatric surgery.	8185 pregnant women with a history of bariatric surgery, 212,945 women with obesity, and 211,155 women without obesity.	N/A	N/A	Retrospective cohort (Conference abstract)	N/A	Mental health disorders are more prevalent in pregnant women with prior bariatric surgery compared to those with obesity and those without obesity.
Jans et al. (2018), Belgium	Examined the impact of bariatric surgery on anxiety and depression in pregnancy, compared to women with untreated obesity.	54 pregnant women with history of bariatric surgery, 25 women with obesity.	Roux-en-Y gastric bypass (*n* = 45, 95.8%)	45.6 ± 29.9 months	Case-Control	State-Trait Anxiety Inventory and Edinburgh Postnatal Depression Scale	Anxiety was higher in women with bariatric surgery compared to women with untreated obesity. In women with bariatric surgery, every 10 kg weight loss after surgery and smoking during pregnancy increased anxiety scores, while every additional hour of sleep during pregnancy correlated with reduced anxiety scores.
Kim et al. (2022), United States	Assessed depression and/or anxiety in pregnancy following bariatric surgery.	1427 pregnant women with history of bariatric surgery, 2854 women with obesity.	N/A	N/A	Case-Control	N/A	The surgery group experienced higher levels of depression and/or anxiety during pregnancy compared to the control group.
Kok et al. (2018), United States	Examined maternal and infant outcomes in pregnancy following bariatric surgery.	91 pregnant women with history of bariatric surgery.	Roux-en-Y gastric bypass (*n* = 70, 77%)	N/A	Observational investigation (Conference abstract)	Positive screenings; Referrals; Antidepressant medication increases; Suicidality; Provider diagnoses	Postnatal depression was observed in 38% of women, significantly higher than the 13–18% reported in the general population of pregnant women with obesity.
Nowak and Da Cunha (2018), Brazil	Described the occurrence of depression in pregnant women with a history of bariatric surgery.	150 pregnant women with history of bariatric surgery	N/A	N/A	Cross-sectional (Conference abstract)	Anxiety and Stress Scale (DASS-21 Scale)	The prevalence of gestational depression was 34.6%. The prevalence of different eating disorders was 42.1%.
Rocha et al. (2022), Brazil	Investigated the prevalence and factors associated with depressive symptoms in pregnant women who had bariatric surgery	247 pregnant women with history of bariatric surgery	Gastric Bypass (*n* = 216, 87.4%)	After 18 months (*n* = 157, 63.6%)	Cohort	Brazilian version of the Depression, Anxiety and Stress Scale (DASS-21)	The prevalence of depressive symptoms was 32.8%. Women who were unmarried or had a psychiatric history were at higher risk of depression. The prevalence of compulsive behaviours was 39.4%.
Walter et al. (2021), Brazil	Evaluation of obstetric and neonatal outcomes in pregnancies after RYGB	132 pregnant women with history of bariatric surgery	Roux-en-Y gastric bypass (*n* = 132, 100%)	16–49 months	Retrospective Cohort	Psychiatric team diagnosis or self-reported postpartum depression	15.2% of mothers underwent psychopharmacological treatment during pregnancy, and 7.6% were diagnosed with postpartum depression.
Yu et al. (2022), United States	Examined the associations between maternal mental health and birth outcomes in pregnant in women with a history of bariatric surgery.	179 pregnancies from 160 pregnant women with history of bariatric surgery	Roux-en-Y gastric bypass (*n* = 128, 71.9%)	3.71 ± 3.16 years	Retrospective Cohort	Patient Health Questionnaire-9; Prescription of medications	Nearly two-thirds of women (64%) had depression/anxiety during pregnancy.

N/A: Not Applicable

All studies were included in the quality appraisal, except for three conference abstracts that lacked sufficient detail. Table 2 shows the percentage of quality criteria met by each study. Most studies addressed over 80% of the quality criteria, with none dropping below 60%, indicating a high level of overall quality across the included studies (JBI, 2020).

**Table 2:** proportion of satisfied criteria met using JBI critical appraisal checklist for studies (*n* = 5)

	Item	1	2	3	4	5	6	7	8	9	10	11	score (%)
Jans et al. (2018)	Prospective Cohort	no	yes	yes	yes	unclear	yes	yes	yes	unclear	unclear	yes	17(77)
Kim et al. (2022)	Case-Control	yes	yes	yes	yes	yes	yes	yes	yes	yes	yes		20(100)
Rocha et al. (2022)	Cross-sectional	yes	yes	yes	yes	yes	yes	yes	yes	N/A	N/A	N/A	16(100)
Walter et al. (2021)	Retrospective Cohort	N/A	N/A	yes	yes	yes	unclear	no	yes	unclear	unclear	yes	13(72)
Yu et al. (2022)	Retrospective Cohort	N/A	N/A	yes	yes	yes	unclear	yes	yes	N/A	N/A	yes	13(92)

*JBI* (Joanna Briggs Institute); Score: Yes: 2. Unclear: 1. No: 0

### Narrative synthesis

#### Prevalence of depression and/or anxiety

The evidence suggests that women who have had bariatric surgery are more likely to have depression and/or anxiety during pregnancy compared to women who have not had bariatric surgery. A total of 6 out of 8 studies reported the prevalence of depression or anxiety during pregnancy. Among these, two studies assessed depression only and four studies assessed depression and/or anxiety. In all these studies, women with a history of bariatric surgery had a higher prevalence of depression and/or anxiety compared with the general pregnancy population or pregnant women without bariatric surgery. In a cohort study, the prevalence of moderate depressive symptoms was 32.8% among pregnant women who had undergone bariatric surgery, with rates reaching 40.6% in the first trimester and 34.3% in the third. The authors mentioned that this rate was higher than the approximately 20% prevalence found in low-risk pregnant women in Brazil (Rocha et al. 2022). Another study conducted in Brazil reported similar findings, indicating a prevalence of 34.6% of depression in pregnant women with a history of bariatric surgery (Nowak and Da Cunha 2018). In a study by Jans et al. (2018), about half of the women who underwent bariatric surgery had higher state and trait anxiety scores during both the first and third trimesters. Anxiety scores were significantly higher for women with bariatric surgery history than those with untreated obesity at both time points. However, no differences between the groups were observed in terms of depression scores. In a case-control study, the prevalence of depression or anxiety was 24.4% in the bariatric surgery group, compared to 14.3% in the control group (*p* < 0.01) (Kim et al. 2022). After adjusting for covariates, the bariatric surgery group had 1.51 times higher odds of experiencing depression and/or anxiety during pregnancy than the control group (OR = 1.51, *p* < 0.01) (Kim et al. 2022). In a retrospective study in Brazil, 20 out of 132 (15.2%) women with a history of bariatric surgery used psychopharmacological treatments during pregnancy, including 2 for bipolar disorder and 18 for depression or anxiety (Walter et al. 2021). In another retrospective study by Yu et al. (2022), nearly two-thirds of the women (64%) with a history of bariatric surgery experienced depression or anxiety during pregnancy. Additionally, Fatima and Popov (2022) highlighted that mental health disorders during pregnancy were more prevalent in women with prior bariatric surgery compared to those with obesity and those without obesity.

Two studies, both conducted in Brazil, reported the prevalence of postpartum depression in women with a history of bariatric surgery, and their results were inconsistent. In a retrospective study, the prevalence of postpartum depression in women with a history of bariatric surgery was 7.6%, which was lower than the general pregnancy population rate of 14% reported in Brazil (Walter et al. 2021). However, in another study, postnatal depression was observed in 38% of women, significantly higher than the 13–18% reported in the general pregnancy population of women with obesity (Kok et al. 2018).

#### Other mental health disorders

Two studies, both conducted in Brazil, reported the prevalence of other mental health disorders, including eating disorders or alcohol/substance use disorders, during pregnancy in women with a history of bariatric surgery. In a cohort study, 32.4% (23 mothers) reported experiencing eating compulsions, which were related to conditions such as bulimia nervosa, anorexia nervosa, and binge eating disorder (Rocha et al. 2022). Additionally, 4.2% (3 mothers) were found to have alcohol or substance use disorders (Rocha et al. 2022). Other compulsions, such as those related to sex, gambling, physical exercise, and shopping, were reported by 2.8% (2 mothers) (Rocha et al. 2022). The study by Nowak and Da Cunha (2018) found that 42.1% of pregnant women with a history of bariatric surgery experienced eating disorders, including anorexia, bulimia, and binge eating.

#### Factors associated with depression and/or anxiety

Several factors were associated with higher levels of anxiety and depression in pregnant women with histories of bariatric surgery. Marital status, psychiatric history, and smoking were significant factors across three studies (Rocha et al. 2022; Jans et al. 2018; Kim et al. 2022). In a cohort study of 247 pregnant women who had bariatric surgery prior to pregnancy, single women were three times more likely to develop depressive symptoms compared to married women, and women with prior psychiatric disorders had more than double the risk of depression (Rocha et al. 2022). On the other hand, planned and desired pregnancies were identified as potential protective factors for mental health, with 41% and 45% lower risks of depression in women with planned and desired pregnancies respectively (Rocha et al. 2022). The results of a case-control study (Jans et al. 2018) highlighted that for pregnant women with histories of bariatric surgery, every 10-kg postoperative weight loss was linked to a rise in anxiety scores in the first trimester. Smoking during pregnancy was also associated with increased anxiety scores for this group of women in the study. On the other hand, every additional hour of sleep in the third trimester was associated with a decrease in anxiety scores (Jans et al. 2018). In another case-control study by Kim et al. (2022), being unmarried, having a psychiatric history, and smoking were linked to increased odds of depression and anxiety during pregnancy for women with histories with bariatric surgery and those without.

In contrast, some studies found that factors such as nutritional deficiency, body mass index (BMI) category at the time of surgery, and the interval between surgery and conception were not significantly associated with mental health outcomes during pregnancy or postpartum. The results of a case-control study highlighted that anxiety and depression scores during pregnancy were not correlated with or explained by inadequate intake of polyunsaturated fatty acids, folate, or vitamin B12 after bariatric surgery (Jans et al. 2018). In a study by Kim et al. (2022), there were no significant differences in the risk of depression or anxiety across various BMI categories at the time of surgery, specifically the ranges of 30–34.9, 35–39.9, 40–44.9, and 45–49.9 kg/m². In a retrospective cohort study aimed at evaluating obstetric and neonatal outcomes in pregnancies following Roux-en-Y gastric bypass (RYGB), the interval between RYGB and conception was categorised into three groups: 0–12 months (early conception), 12–47 months, and ≥ 48 months (late conception). The prevalence of psychopharmacological treatment use during pregnancy or postpartum depression was found to be similar across all three groups (Walter et al. 2021).

## Discussion

To our knowledge, this scoping review is the first study to summarise the impact of bariatric surgery on mental health during pregnancy and the postpartum period. The evidence shows that women who have had bariatric surgery are more likely to have depression and/or anxiety during pregnancy than women without bariatric surgery. Although there has been a significant increase in bariatric surgeries since 2008, particularly among women of reproductive age (Mokhlesi et al. 2024; Snoek et al. 2021), research on mental health during pregnancy and postpartum following bariatric surgery is notably lacking. Our review includes eight studies, three of which were conference abstracts. All of these studies focussed on depression and anxiety, while other mental health disorders during pregnancy and postpartum were subject to limited investigation. Overall, the included studies were of high-quality, however, their limited number and narrow focus highlight the need for further research to comprehensively assess the mental health outcomes of this population during pregnancy. Another gap identified in the review is that only two studies, both conducted in Brazil, addressed postpartum depression in mothers with a history of bariatric surgery. The results were inconsistent; one study found a lower rate of postpartum depression compared to the general pregnancy population, while the other found a higher rate. A possible explanation for this discrepancy could be the use of different diagnostic criteria or assessment methods for postpartum depression in each study. Therefore, further research with larger sample sizes and comparison groups is needed to better understand the relationship between bariatric surgery and postpartum mental health.

The review revealed that, in case-control studies, the prevalence of anxiety and depression during pregnancy was higher among women who had undergone bariatric surgery compared to women with or without obesity or those who had not had bariatric surgery. Additionally, studies without comparable groups indicated that these mental health issues were even more prevalent than in the general pregnancy population. The physiological and psychological changes that occur during pregnancy, combined with the effects of bariatric surgery, may interact and raise the risk of depression and anxiety this group of women (Kim et al. 2022). Depression and anxiety disorders are the most common pre-surgery mental health disorders among bariatric surgery recipients. In a study by Kim et al. (2022), women in the bariatric surgery group were about twice as likely to have a diagnosis of depression and/or anxiety in the 12 months before pregnancy compared to women with untreated obesity in the control group. Although bariatric surgery may improve anxiety and depressive symptoms in the short term, long-term studies show that these mental health disorders return to pre-surgery levels four and nine years after the procedure (Law et al. 2023). On the other hand, bariatric surgery can be seen as a source of stress and may trigger mental health issues in individuals with existing psychological vulnerabilities (Ratcliffe 2021). During pregnancy, the situation can become even more complicated. Bariatric surgery leads to both physical and psychological changes that can make it challenging for mothers to adapt to the bodily and psychological changes associated with pregnancy (Bąk-Sosnowska and Naworska 2023). It is important that clinicians recognise the link between bariatric surgery history and increased risk for mental health disorders during pregnancy in order to provide preventative and comprehensive care to support the mental health needs of these women. For women with histories of bariatric surgery, having awareness of the increased risks of depression and anxiety during pregnancy can empower them to monitor their mental health. This awareness may encourage women to engage in preventive or therapeutic strategies during pregnancy and the postpartum period.

Our review found that being single, having psychiatric history, and smoking are associated with increased rates of depression and anxiety in pregnant women who have undergone bariatric surgery. These findings are consistent with other studies that identify similar risk factors for mental disorders during pregnancy. A systematic review of 57 studies examining risk factors for antenatal depression in the general pregnant population highlighted that being single, having pre-existing mental health disorders, smoking, and low socioeconomic status were all factors linked to a higher risk of depression during pregnancy (Lancaster et al. 2010). Single mothers may need emotional and social support systems to help them cope with the challenges that the pregnancy and postpartum period can bring (Rocha et al. 2022). It is likely that support is even more important for unpartnered pregnant women who have had bariatric surgery (Sweet and Vasilevski 2022). The higher prevalence of anxiety among pregnant women who smoke may be due to the nervous system effects of nicotine (Silva et al. 2017). Concerns about weight regain after surgery are often linked to mental health issues in people who have undergone bariatric surgery (Law et al. 2023), however, only one study examined this factor in pregnant women after bariatric surgery (Jans et al. 2018). The study found that anxiety scores were higher in women who experienced greater postoperative weight loss (Jans et al. 2018). The authors suggested that the expected weight gain during pregnancy may cause anxiety in women who have achieved significant long-term weight loss after bariatric surgery pre-pregnancy (Jans et al. 2018).

### Strengths and limitations

This article is the first review of studies to summarise the impact of bariatric surgery on mental health during pregnancy and the postpartum period. Two reviewers independently assessed all relevant studies, with a third available for conflict resolution, ensuring a thorough evaluation. The review followed PRISMA-ScR guidelines, and quality appraisal was performed using the JBI tools, which enhanced the transparency and interpretability of findings. However, several limitations exist. Non-English studies were excluded due to language barriers, and reliance on specific databases could have omitted relevant research. Additionally, conference abstracts from three out of eight studies were only available, limiting the amount of data accessible to draw conclusions. Finally, the small number of included studies, the limited range of psychological diagnoses reported, and heterogeneity in study designs reduced the strength of the conclusions that could be drawn from this review.

## Conclusion

The findings of this scoping review indicated that women who have undergone bariatric surgeries may be at increased risk of experiencing depression and anxiety during pregnancy than women who do not have bariatric surgery. The studies revealed limited and inconsistent findings regarding postpartum depression and other mental health disorders. These insights suggest that further investigation is needed, however the limited evidence suggests that mental health screening and support for women with histories of bariatric surgery before, during, and after pregnancy could be beneficial.

## Electronic Supplementary Material

Below is the link to the electronic supplementary material.

Supplementary Material 1

## Data Availability

No datasets were generated or analysed during the current study.
