# Supplementary material for: Mental health during pregnancy and postpartum in women with a history of bariatric surgery: A scoping review
Source: Arch Womens Ment Health. 2026 May 19;29(3):83. doi: 10.1007/s00737-026-01727-w (PMC13186867; doi:10.1007/s00737-026-01727-w)
Supplement: Supplementary file 1 — Supplementary Material 1 [file 737_2026_1727_MOESM1_ESM.pdf]

EBSCOhost

Mon, December 23, 2024 10:47:49 AM

#	Query	Limiters/Expanders	Last Run Via	Results
S164	S113 AND S134 AND S163	Search modes - Proximity	Interface - EBSCOhost Research Databases<br>Search Screen - Advanced Search<br>Database - Academic Search Complete;AgeLine;AMED - The Allied and Complementary Medicine Database;Applied Science & Technology Source;Art & Architecture Source;Avery Index to Architectural Periodicals;Business Source Complete;CINAHL Complete;Communication & Mass Media Complete;Criminal Justice Abstracts with Full Text;eBook Academic Collection (EBSCOhost);eBook Collection (EBSCOhost);EconLit;Education Source;E-Journals;Environment Complete;ERIC;European Views of the Americas: 1493 to 1750;Film & Television Literature Index with Full Text;Global Health;GreenFILE;Health Business Elite;Health Policy Reference Center;Health Source - Consumer Edition;Health Source: Nursing/Academic Edition;Historical Abstracts with Full Text;Humanities Source;International Bibliography of Theatre &	34

			Dance with Full Text;Legal Source;LGBTQ+ Source;Library & Information Science Source;Library, Information Science & Technology Abstracts;MAS Ultra - School Edition;MasterFILE Premier;MEDLINE Complete;Mental Measurements Yearbook;MLA Directory of Periodicals;MLA International Bibliography;Newspaper Source Plus;Newswires;Philosopher's Index;Political Science Complete;APA PsycArticles;APA PsycBooks;APA PsycExtra;Psychology and Behavioral Sciences Collection;APA PsycInfo;APA PsycTests;Regional Business News;Religion and Philosophy Collection;Social Work Abstracts;SocINDEX with Full Text;SPORTDiscus with Full Text;OpenDissertations	
S163	S135 OR S136 OR S137 OR S138 OR S139 OR S140 OR S141 OR S142 OR S143 OR S144 OR S145 OR S146 OR S147 OR S148 OR S149 OR S150 OR S151 OR S152 OR S153 OR S154 OR S155 OR S156 OR S157 OR S158 OR S159 OR S160 OR S161 OR S162	Search modes - Proximity	Interface - EBSCOhost Research Databases<br>Search Screen - Advanced Search<br>Database - MEDLINE Complete	Display
S162	(MH "Pregnancy+")	Search modes - Proximity	Interface - EBSCOhost Research Databases<br>Search Screen - Advanced Search<br>Database - MEDLINE Complete	Display

S161	(MH "Prenatal Diagnosis+")	Search modes - Proximity	Interface - EBSCOhost Research Databases<br>Search Screen - Advanced Search<br>Database - MEDLINE Complete	Display
S160	(MH "Prenatal Care")	Search modes - Proximity	Interface - EBSCOhost Research Databases<br>Search Screen - Advanced Search<br>Database - MEDLINE Complete	Display
S159	(MH "Postpartum Period+")	Search modes - Proximity	Interface - EBSCOhost Research Databases<br>Search Screen - Advanced Search<br>Database - MEDLINE Complete	Display
S158	(MM "Postnatal Care")	Search modes - Proximity	Interface - EBSCOhost Research Databases<br>Search Screen - Advanced Search<br>Database - MEDLINE Complete	Display
S157	(MH "Perinatal Care+")	Search modes - Proximity	Interface - EBSCOhost Research Databases<br>Search Screen - Advanced Search<br>Database - MEDLINE Complete	Display
S156	AB Peri partum OR TI Peri partum	Search modes - Proximity	Interface - EBSCOhost Research Databases<br>Search Screen - Advanced Search<br>Database - MEDLINE Complete	Display
S155	AB Peripartum OR TI Peripartum	Search modes - Proximity	Interface - EBSCOhost Research Databases<br>Search Screen - Advanced Search<br>Database - MEDLINE Complete	Display

S154	AB Peri-partum OR TI Peri-partum	Search modes - Proximity	Interface - EBSCOhost Research Databases<br>Search Screen - Advanced Search<br>Database - MEDLINE Complete	Display
S153	AB pre natal OR TI pre natal	Search modes - Proximity	Interface - EBSCOhost Research Databases<br>Search Screen - Advanced Search<br>Database - MEDLINE Complete	Display
S152	AB pre-natal OR TI pre-natal	Search modes - Proximity	Interface - EBSCOhost Research Databases<br>Search Screen - Advanced Search<br>Database - MEDLINE Complete	Display
S151	AB prenatal OR TI prenatal	Search modes - Proximity	Interface - EBSCOhost Research Databases<br>Search Screen - Advanced Search<br>Database - MEDLINE Complete	Display
S150	AB ante natal OR TI ante natal	Search modes - Proximity	Interface - EBSCOhost Research Databases<br>Search Screen - Advanced Search<br>Database - MEDLINE Complete	Display
S149	AB ante-natal OR TI ante-natal	Search modes - Proximity	Interface - EBSCOhost Research Databases<br>Search Screen - Advanced Search<br>Database - MEDLINE Complete	Display
S148	AB antenatal OR TI antenatal	Search modes - Proximity	Interface - EBSCOhost Research Databases<br>Search Screen - Advanced Search<br>Database - MEDLINE Complete	Display

S147	AB ante partum OR TI ante partum	Search modes - Proximity	Interface - EBSCOhost Research Databases<br>Search Screen - Advanced Search<br>Database - MEDLINE Complete	Display
S146	AB ante-partum OR TI ante-partum	Search modes - Proximity	Interface - EBSCOhost Research Databases<br>Search Screen - Advanced Search<br>Database - MEDLINE Complete	Display
S145	AB antepartum OR TI antepartum	Search modes - Proximity	Interface - EBSCOhost Research Databases<br>Search Screen - Advanced Search<br>Database - MEDLINE Complete	Display
S144	AB puerp* OR TI puerp*	Search modes - Proximity	Interface - EBSCOhost Research Databases<br>Search Screen - Advanced Search<br>Database - MEDLINE Complete	Display
S143	AB peri natal* OR TI peri natal*	Search modes - Proximity	Interface - EBSCOhost Research Databases<br>Search Screen - Advanced Search<br>Database - MEDLINE Complete	Display
S142	AB peri-natal* OR TI peri-natal*	Search modes - Proximity	Interface - EBSCOhost Research Databases<br>Search Screen - Advanced Search<br>Database - MEDLINE Complete	Display
S141	AB perinatal* OR TI perinatal*	Search modes - Proximity	Interface - EBSCOhost Research Databases<br>Search Screen - Advanced Search<br>Database - MEDLINE Complete	Display

S140	AB post natal* OR TI post natal*	Search modes - Proximity	Interface - EBSCOhost Research Databases<br>Search Screen - Advanced Search<br>Database - MEDLINE Complete	Display
S139	AB post-natal* OR TI post-natal*	Search modes - Proximity	Interface - EBSCOhost Research Databases<br>Search Screen - Advanced Search<br>Database - MEDLINE Complete	Display
S138	AB postnatal* OR TI postnatal*	Search modes - Proximity	Interface - EBSCOhost Research Databases<br>Search Screen - Advanced Search<br>Database - MEDLINE Complete	Display
S137	AB post partum OR TI post partum	Search modes - Proximity	Interface - EBSCOhost Research Databases<br>Search Screen - Advanced Search<br>Database - MEDLINE Complete	Display
S136	AB post-partum OR TI post-partum	Search modes - Proximity	Interface - EBSCOhost Research Databases<br>Search Screen - Advanced Search<br>Database - MEDLINE Complete	Display
S135	AB postpartum OR TI postpartum	Search modes - Proximity	Interface - EBSCOhost Research Databases<br>Search Screen - Advanced Search<br>Database - MEDLINE Complete	Display
S134	S114 OR S115 OR S116 OR S117 OR S118 OR S119 OR S120 OR S121 OR S122 OR S123 OR S124 OR S125 OR S126 OR S127 OR S128 OR S129 OR S130 OR S131 OR S132 OR S133	Search modes - Proximity	Interface - EBSCOhost Research Databases<br>Search Screen - Advanced Search<br>Database - MEDLINE Complete	Display
S133	(MM "Gastroplasty")	Search modes - Proximity	Interface - EBSCOhost Research Databases	Display

			Search Screen - Advanced Search<br>Database - MEDLINE Complete	
S132	AB "Gastric Band*" OR TI "Gastric Band*"	Search modes - Proximity	Interface - EBSCOhost Research Databases<br>Search Screen - Advanced Search<br>Database - MEDLINE Complete	Display
S131	(MM "Body Weight Maintenance")	Search modes - Proximity	Interface - EBSCOhost Research Databases<br>Search Screen - Advanced Search<br>Database - MEDLINE Complete	Display
S130	(MH "Obesity Management+")	Search modes - Proximity	Interface - EBSCOhost Research Databases<br>Search Screen - Advanced Search<br>Database - MEDLINE Complete	Display
S129	(MH "Gastrectomy+")	Search modes - Proximity	Interface - EBSCOhost Research Databases<br>Search Screen - Advanced Search<br>Database - MEDLINE Complete	Display
S128	(MM "Gastric Balloon")	Search modes - Proximity	Interface - EBSCOhost Research Databases<br>Search Screen - Advanced Search<br>Database - MEDLINE Complete	Display
S127	(MM "Gastric Bypass")	Search modes - Proximity	Interface - EBSCOhost Research Databases<br>Search Screen - Advanced Search<br>Database - MEDLINE Complete	Display
S126	(MH "Bariatrics+")	Search modes - Proximity	Interface - EBSCOhost Research Databases<br>Search Screen - Advanced Search<br>Database - MEDLINE Complete	Display

S125	(MH "Bariatric Surgery+")	Search modes - Proximity	Interface - EBSCOhost Research Databases<br>Search Screen - Advanced Search<br>Database - MEDLINE Complete	Display
S124	AB gastroplasty OR TI gastroplasty	Search modes - Proximity	Interface - EBSCOhost Research Databases<br>Search Screen - Advanced Search<br>Database - MEDLINE Complete	Display
S123	AB "Gastric sleeve" OR TI "Gastric sleeve"	Search modes - Proximity	Interface - EBSCOhost Research Databases<br>Search Screen - Advanced Search<br>Database - MEDLINE Complete	Display
S122	AB "Roux-en-Y Gastric Bypass" OR TI "Roux-en-Y Gastric Bypass"	Search modes - Proximity	Interface - EBSCOhost Research Databases<br>Search Screen - Advanced Search<br>Database - MEDLINE Complete	Display
S121	AB "Lap Gastric Band*" OR TI "Lap Gastric Band*"	Search modes - Proximity	Interface - EBSCOhost Research Databases<br>Search Screen - Advanced Search<br>Database - MEDLINE Complete	Display
S120	AB "Stomach Stapling" OR TI "Stomach Stapling"	Search modes - Proximity	Interface - EBSCOhost Research Databases<br>Search Screen - Advanced Search<br>Database - MEDLINE Complete	Display
S119	AB "Metabolic Surg*" OR TI "Metabolic Surg*"	Search modes - Proximity	Interface - EBSCOhost Research Databases<br>Search Screen - Advanced Search<br>Database - MEDLINE Complete	Display

S118	AB "Maternal weight loss" OR TI "Maternal weight loss"	Search modes - Proximity	Interface - EBSCOhost Research Databases<br>Search Screen - Advanced Search<br>Database - MEDLINE Complete	Display
S117	AB "Weight loss surg*" OR TI "Weight loss surg*"	Search modes - Proximity	Interface - EBSCOhost Research Databases<br>Search Screen - Advanced Search<br>Database - MEDLINE Complete	Display
S116	AB "Obesity surg*" OR TI "Obesity surg*"	Search modes - Proximity	Interface - EBSCOhost Research Databases<br>Search Screen - Advanced Search<br>Database - MEDLINE Complete	Display
S115	AB Bariatric* OR TI Bariatric*	Search modes - Proximity	Interface - EBSCOhost Research Databases<br>Search Screen - Advanced Search<br>Database - MEDLINE Complete	Display
S114	AB "Bariatric* surg*" OR TI "Bariatric* surg*"	Search modes - Proximity	Interface - EBSCOhost Research Databases<br>Search Screen - Advanced Search<br>Database - MEDLINE Complete	Display
S113	S103 OR S104 OR S105 OR S106 OR S107 OR S108 OR S109 OR S110 OR S111 OR S112	Search modes - Proximity	Interface - EBSCOhost Research Databases<br>Search Screen - Advanced Search<br>Database - MEDLINE Complete	Display
S112	(MH "Mood Disorders+")	Search modes - Proximity	Interface - EBSCOhost Research Databases<br>Search Screen - Advanced Search<br>Database - MEDLINE Complete	Display

S111	(MH "Mental Disorders+")	Search modes - Proximity	Interface - EBSCOhost Research Databases<br>Search Screen - Advanced Search<br>Database - MEDLINE Complete	Display
S110	(MH "Anxiety Disorders+")	Search modes - Proximity	Interface - EBSCOhost Research Databases<br>Search Screen - Advanced Search<br>Database - MEDLINE Complete	Display
S109	(MH "Anxiety+")	Search modes - Proximity	Interface - EBSCOhost Research Databases<br>Search Screen - Advanced Search<br>Database - MEDLINE Complete	Display
S108	(MM "Depression, Postpartum")	Search modes - Proximity	Interface - EBSCOhost Research Databases<br>Search Screen - Advanced Search<br>Database - MEDLINE Complete	Display
S107	(MH "Depressive Disorder+")	Search modes - Proximity	Interface - EBSCOhost Research Databases<br>Search Screen - Advanced Search<br>Database - MEDLINE Complete	Display
S106	(MM "Depression")	Search modes - Proximity	Interface - EBSCOhost Research Databases<br>Search Screen - Advanced Search<br>Database - MEDLINE Complete	Display
S105	(MH "Stress, Psychological+")	Search modes - Proximity	Interface - EBSCOhost Research Databases<br>Search Screen - Advanced Search<br>Database - MEDLINE Complete	Display

S104	(MM "Mental Health")	Search modes - Proximity	Interface - EBSCOhost Research Databases<br>Search Screen - Advanced Search<br>Database - MEDLINE Complete	Display
S103	S1 OR S2 OR S3 OR S4 OR S5 OR S6 OR S7 OR S8 OR S9 OR S10 OR S11 OR S12 OR S13 OR S14 OR S15 OR S16 OR S17 OR S18 OR S19 OR S20 OR S21 OR S22 OR S23 OR S24 OR S25 OR S26 OR S27 OR S28 OR S29 OR S30 OR S31 OR S32 OR S33 OR S34 OR S35 OR S36 OR S37 OR S38 OR S39 OR S40 OR S41 OR S42 OR S43 OR S44 OR S45 OR S46 OR S47 OR S48 OR S49 OR S50 OR S51 OR S52 OR S53 OR S54 OR S55 OR S56 OR S57 OR S58 OR S59 OR S60 OR S61 OR S62 OR S63 OR S64 OR S65 OR S66 OR S67 OR S68 OR S69 OR S70 OR S71 OR S72 OR S73 OR S74 OR S75 OR S76 OR S77 OR S78 OR S79 OR S80 OR S81 OR S82 OR S83 OR S84 OR S85 OR S86 OR S87 OR S88 OR S89 OR S90 OR S91 OR S92 OR S93 OR S94 OR S95 OR S96 OR S97 OR S98 OR S99 OR S100 OR S101 OR S102	Search modes - Proximity	Interface - EBSCOhost Research Databases<br>Search Screen - Advanced Search<br>Database - MEDLINE Complete	Display
S102	AB Depressive* OR TI Depressive*	Search modes - Proximity	Interface - EBSCOhost Research Databases<br>Search Screen - Advanced	Display

			Search<br>Database - MEDLINE Complete	
S101	AB Depression* OR TI Depression*	Search modes - Proximity	Interface - EBSCOhost Research Databases<br>Search Screen - Advanced Search<br>Database - MEDLINE Complete	Display
S100	AB "Emotional well-being" OR TI "Emotional well-being"	Search modes - Proximity	Interface - EBSCOhost Research Databases<br>Search Screen - Advanced Search<br>Database - MEDLINE Complete	Display
S99	AB "Postpartum depression*" OR TI "Postpartum depression*"	Search modes - Proximity	Interface - EBSCOhost Research Databases<br>Search Screen - Advanced Search<br>Database - MEDLINE Complete	Display
S98	AB "Mood disorder*" OR TI "Mood disorder*"	Search modes - Proximity	Interface - EBSCOhost Research Databases<br>Search Screen - Advanced Search<br>Database - MEDLINE Complete	Display
S97	AB "behavior* disorder*" OR TI "behavior* disorder*"	Search modes - Proximity	Interface - EBSCOhost Research Databases<br>Search Screen - Advanced Search<br>Database - MEDLINE Complete	Display
S96	AB "behaviour* disorder*" OR TI "behaviour* disorder*"	Search modes - Proximity	Interface - EBSCOhost Research Databases<br>Search Screen - Advanced Search<br>Database - MEDLINE Complete	Display
S95	AB Psychiatry* OR TI Psychiatry*	Search modes - Proximity	Interface - EBSCOhost Research Databases<br>Search Screen - Advanced Search<br>Database - MEDLINE Complete	Display
S94	AB Psychology* OR TI Psychology*	Search modes - Proximity	Interface - EBSCOhost Research Databases	Display

			Search Screen - Advanced Search<br>Database - MEDLINE Complete	
S93	AB "psychology* health" OR TI "psychology* health"	Search modes - Proximity	Interface - EBSCOhost Research Databases<br>Search Screen - Advanced Search<br>Database - MEDLINE Complete	Display
S92	AB "psychology* impairment*" OR TI "psychology* impairment*"	Search modes - Proximity	Interface - EBSCOhost Research Databases<br>Search Screen - Advanced Search<br>Database - MEDLINE Complete	Display
S91	AB "psychology* issue*" OR TI "psychology* issue*"	Search modes - Proximity	Interface - EBSCOhost Research Databases<br>Search Screen - Advanced Search<br>Database - MEDLINE Complete	Display
S90	AB "psychology* distress" OR TI "psychology* distress"	Search modes - Proximity	Interface - EBSCOhost Research Databases<br>Search Screen - Advanced Search<br>Database - MEDLINE Complete	Display
S89	AB "psychology* crisis" OR TI "psychology* crisis"	Search modes - Proximity	Interface - EBSCOhost Research Databases<br>Search Screen - Advanced Search<br>Database - MEDLINE Complete	Display
S88	AB "psychology* problem*" OR TI "psychology* problem*"	Search modes - Proximity	Interface - EBSCOhost Research Databases<br>Search Screen - Advanced Search<br>Database - MEDLINE Complete	Display
S87	AB "psychology* illness*" OR TI "psychology* illness*"	Search modes - Proximity	Interface - EBSCOhost Research Databases<br>Search Screen - Advanced Search<br>Database - MEDLINE Complete	Display

S86	AB "psychology* ill" OR TI "psychology* ill"	Search modes - Proximity	Interface - EBSCOhost Research Databases<br>Search Screen - Advanced Search<br>Database - MEDLINE Complete	Display
S85	AB "psychology* disease*" OR TI "psychology* disease*"	Search modes - Proximity	Interface - EBSCOhost Research Databases<br>Search Screen - Advanced Search<br>Database - MEDLINE Complete	Display
S84	AB "psychology* disorder*" OR TI "psychology* disorder*"	Search modes - Proximity	Interface - EBSCOhost Research Databases<br>Search Screen - Advanced Search<br>Database - MEDLINE Complete	Display
S83	AB "psychiatry* health" OR TI "psychiatry* health"	Search modes - Proximity	Interface - EBSCOhost Research Databases<br>Search Screen - Advanced Search<br>Database - MEDLINE Complete	Display
S82	AB "psychiatry* impairment*" OR TI "psychiatry* impairment*"	Search modes - Proximity	Interface - EBSCOhost Research Databases<br>Search Screen - Advanced Search<br>Database - MEDLINE Complete	Display
S81	AB "psychiatry* issue*" OR TI "psychiatry* issue*"	Search modes - Proximity	Interface - EBSCOhost Research Databases<br>Search Screen - Advanced Search<br>Database - MEDLINE Complete	Display
S80	AB "psychiatry* distress" OR TI "psychiatry* distress"	Search modes - Proximity	Interface - EBSCOhost Research Databases<br>Search Screen - Advanced Search<br>Database - MEDLINE Complete	Display

S79	AB "psychiatry* crisis" OR TI "psychiatry* crisis"	Search modes - Proximity	Interface - EBSCOhost Research Databases<br>Search Screen - Advanced Search<br>Database - MEDLINE Complete	Display
S78	AB "psychiatry* problem*" OR TI "psychiatry* problem*"	Search modes - Proximity	Interface - EBSCOhost Research Databases<br>Search Screen - Advanced Search<br>Database - MEDLINE Complete	Display
S77	AB "psychiatry* illness*" OR TI "psychiatry* illness*"	Search modes - Proximity	Interface - EBSCOhost Research Databases<br>Search Screen - Advanced Search<br>Database - MEDLINE Complete	Display
S76	AB "psychiatry* ill" OR TI "psychiatry* ill"	Search modes - Proximity	Interface - EBSCOhost Research Databases<br>Search Screen - Advanced Search<br>Database - MEDLINE Complete	Display
S75	AB "psychiatry* disease*" OR TI "psychiatry* disease*"	Search modes - Proximity	Interface - EBSCOhost Research Databases<br>Search Screen - Advanced Search<br>Database - MEDLINE Complete	Display
S74	AB "psychiatry* disorder*" OR TI "psychiatry* disorder*"	Search modes - Proximity	Interface - EBSCOhost Research Databases<br>Search Screen - Advanced Search<br>Database - MEDLINE Complete	Display
S73	AB "mental* health" OR TI "mental* health"	Search modes - Proximity	Interface - EBSCOhost Research Databases<br>Search Screen - Advanced Search<br>Database - MEDLINE Complete	Display

S72	AB "mental* impairment*" OR TI "mental* impairment*"	Search modes - Proximity	Interface - EBSCOhost Research Databases<br>Search Screen - Advanced Search<br>Database - MEDLINE Complete	Display
S71	AB "mental* issue*" OR TI "mental* issue*"	Search modes - Proximity	Interface - EBSCOhost Research Databases<br>Search Screen - Advanced Search<br>Database - MEDLINE Complete	Display
S70	AB "mental* distress" OR TI "mental* distress"	Search modes - Proximity	Interface - EBSCOhost Research Databases<br>Search Screen - Advanced Search<br>Database - MEDLINE Complete	Display
S69	AB "mental* crisis" OR TI "mental* crisis"	Search modes - Proximity	Interface - EBSCOhost Research Databases<br>Search Screen - Advanced Search<br>Database - MEDLINE Complete	Display
S68	AB "mental* problem*" OR TI "mental* problem*"	Search modes - Proximity	Interface - EBSCOhost Research Databases<br>Search Screen - Advanced Search<br>Database - MEDLINE Complete	Display
S67	AB "mental* illness*" OR TI "mental* illness*"	Search modes - Proximity	Interface - EBSCOhost Research Databases<br>Search Screen - Advanced Search<br>Database - MEDLINE Complete	Display
S66	AB "mental* ill" OR TI "mental* ill"	Search modes - Proximity	Interface - EBSCOhost Research Databases<br>Search Screen - Advanced Search<br>Database - MEDLINE Complete	Display

S65	AB "mental* disorder*" OR TI "mental* disorder*"	Search modes - Proximity	Interface - EBSCOhost Research Databases<br>Search Screen - Advanced Search<br>Database - MEDLINE Complete	Display
S64	AB "mental* disease*" OR TI "mental* disease*"	Search modes - Proximity	Interface - EBSCOhost Research Databases<br>Search Screen - Advanced Search<br>Database - MEDLINE Complete	Display
S63	AB "psychiatry* disease*" OR TI "psychiatry* disease*"	Search modes - Proximity	Interface - EBSCOhost Research Databases<br>Search Screen - Advanced Search<br>Database - MEDLINE Complete	Display
S62	AB "psychiatry* disorder*" OR TI "psychiatry* disorder*"	Search modes - Proximity	Interface - EBSCOhost Research Databases<br>Search Screen - Advanced Search<br>Database - MEDLINE Complete	Display
S61	AB "mental* health" OR TI "mental* health"	Search modes - Proximity	Interface - EBSCOhost Research Databases<br>Search Screen - Advanced Search<br>Database - MEDLINE Complete	Display
S60	AB "mental* impairment*" OR TI "mental* impairment*"	Search modes - Proximity	Interface - EBSCOhost Research Databases<br>Search Screen - Advanced Search<br>Database - MEDLINE Complete	Display
S59	AB "mental* issue*" OR TI "mental* issue*"	Search modes - Proximity	Interface - EBSCOhost Research Databases<br>Search Screen - Advanced Search<br>Database - MEDLINE Complete	Display

S58	AB "mental* distress" OR TI "mental* distress"	Search modes - Proximity	Interface - EBSCOhost Research Databases<br>Search Screen - Advanced Search<br>Database - MEDLINE Complete	Display
S57	AB "mental* crisis" OR TI "mental* crisis"	Search modes - Proximity	Interface - EBSCOhost Research Databases<br>Search Screen - Advanced Search<br>Database - MEDLINE Complete	Display
S56	AB "mental* problem*" OR TI "mental* problem*"	Search modes - Proximity	Interface - EBSCOhost Research Databases<br>Search Screen - Advanced Search<br>Database - MEDLINE Complete	Display
S55	AB "mental* illness*" OR TI "mental* illness*"	Search modes - Proximity	Interface - EBSCOhost Research Databases<br>Search Screen - Advanced Search<br>Database - MEDLINE Complete	Display
S54	AB "mental* ill" OR TI "mental* ill"	Search modes - Proximity	Interface - EBSCOhost Research Databases<br>Search Screen - Advanced Search<br>Database - MEDLINE Complete	Display
S53	AB "mental* disorder*" OR TI "mental* disorder*"	Search modes - Proximity	Interface - EBSCOhost Research Databases<br>Search Screen - Advanced Search<br>Database - MEDLINE Complete	Display
S52	AB "mental* disease*" OR TI "mental* disease*"	Search modes - Proximity	Interface - EBSCOhost Research Databases<br>Search Screen - Advanced Search<br>Database - MEDLINE Complete	Display

S51	AB Depressive* OR TI Depressive*	Search modes - Proximity	Interface - EBSCOhost Research Databases<br>Search Screen - Advanced Search<br>Database - MEDLINE Complete	Display
S50	AB Depression* OR TI Depression*	Search modes - Proximity	Interface - EBSCOhost Research Databases<br>Search Screen - Advanced Search<br>Database - MEDLINE Complete	Display
S49	AB "Emotional well-being" OR TI "Emotional well-being"	Search modes - Proximity	Interface - EBSCOhost Research Databases<br>Search Screen - Advanced Search<br>Database - MEDLINE Complete	Display
S48	AB "Postpartum depression*" OR TI "Postpartum depression*"	Search modes - Proximity	Interface - EBSCOhost Research Databases<br>Search Screen - Advanced Search<br>Database - MEDLINE Complete	Display
S47	AB "Mood disorder*" OR TI "Mood disorder*"	Search modes - Proximity	Interface - EBSCOhost Research Databases<br>Search Screen - Advanced Search<br>Database - MEDLINE Complete	Display
S46	AB "behavior* disorder*" OR TI "behavior* disorder*"	Search modes - Proximity	Interface - EBSCOhost Research Databases<br>Search Screen - Advanced Search<br>Database - MEDLINE Complete	Display
S45	AB "behaviour* disorder*" OR TI "behaviour* disorder*"	Search modes - Proximity	Interface - EBSCOhost Research Databases<br>Search Screen - Advanced Search<br>Database - MEDLINE Complete	Display

S44	AB Psychiatry* OR TI Psychiatry*	Search modes - Proximity	Interface - EBSCOhost Research Databases<br>Search Screen - Advanced Search<br>Database - MEDLINE Complete	Display
S43	AB Psychology* OR TI Psychology*	Search modes - Proximity	Interface - EBSCOhost Research Databases<br>Search Screen - Advanced Search<br>Database - MEDLINE Complete	Display
S42	AB "psychology* health" OR TI "psychology* health"	Search modes - Proximity	Interface - EBSCOhost Research Databases<br>Search Screen - Advanced Search<br>Database - MEDLINE Complete	Display
S41	AB "psychology* impairment*" OR TI "psychology* impairment*"	Search modes - Proximity	Interface - EBSCOhost Research Databases<br>Search Screen - Advanced Search<br>Database - MEDLINE Complete	Display
S40	AB "psychology* issue*" OR TI "psychology* issue*"	Search modes - Proximity	Interface - EBSCOhost Research Databases<br>Search Screen - Advanced Search<br>Database - MEDLINE Complete	Display
S39	AB "psychology* distress" OR TI "psychology* distress"	Search modes - Proximity	Interface - EBSCOhost Research Databases<br>Search Screen - Advanced Search<br>Database - MEDLINE Complete	Display
S38	AB "psychology* crisis" OR TI "psychology* crisis"	Search modes - Proximity	Interface - EBSCOhost Research Databases<br>Search Screen - Advanced Search<br>Database - MEDLINE Complete	Display

S37	AB "psychology* problem*" OR TI "psychology* problem*"	Search modes - Proximity	Interface - EBSCOhost Research Databases<br>Search Screen - Advanced Search<br>Database - MEDLINE Complete	Display
S36	AB "psychology* illness*" OR TI "psychology* illness*"	Search modes - Proximity	Interface - EBSCOhost Research Databases<br>Search Screen - Advanced Search<br>Database - MEDLINE Complete	Display
S35	AB "psychology* ill" OR TI "psychology* ill"	Search modes - Proximity	Interface - EBSCOhost Research Databases<br>Search Screen - Advanced Search<br>Database - MEDLINE Complete	Display
S34	AB "psychology* disease*" OR TI "psychology* disease*"	Search modes - Proximity	Interface - EBSCOhost Research Databases<br>Search Screen - Advanced Search<br>Database - MEDLINE Complete	Display
S33	AB "psychology* disorder*" OR TI "psychology* disorder*"	Search modes - Proximity	Interface - EBSCOhost Research Databases<br>Search Screen - Advanced Search<br>Database - MEDLINE Complete	Display
S32	AB "psychiatry* health" OR TI "psychiatry* health"	Search modes - Proximity	Interface - EBSCOhost Research Databases<br>Search Screen - Advanced Search<br>Database - MEDLINE Complete	Display
S31	AB "psychiatry* impairment*" OR TI "psychiatry* impairment*"	Search modes - Proximity	Interface - EBSCOhost Research Databases<br>Search Screen - Advanced Search<br>Database - MEDLINE Complete	Display

S30	AB "psychiatry* issue*" OR TI "psychiatry* issue*"	Search modes - Proximity	Interface - EBSCOhost Research Databases<br>Search Screen - Advanced Search<br>Database - MEDLINE Complete	Display
S29	AB "psychiatry* distress" OR TI "psychiatry* distress"	Search modes - Proximity	Interface - EBSCOhost Research Databases<br>Search Screen - Advanced Search<br>Database - MEDLINE Complete	Display
S28	AB "psychiatry* crisis" OR TI "psychiatry* crisis"	Search modes - Proximity	Interface - EBSCOhost Research Databases<br>Search Screen - Advanced Search<br>Database - MEDLINE Complete	Display
S27	AB "psychiatry* problem*" OR TI "psychiatry* problem*"	Search modes - Proximity	Interface - EBSCOhost Research Databases<br>Search Screen - Advanced Search<br>Database - MEDLINE Complete	Display
S26	AB "psychiatry* illness*" OR TI "psychiatry* illness*"	Search modes - Proximity	Interface - EBSCOhost Research Databases<br>Search Screen - Advanced Search<br>Database - MEDLINE Complete	Display
S25	AB "psychiatry* ill" OR TI "psychiatry* ill"	Search modes - Proximity	Interface - EBSCOhost Research Databases<br>Search Screen - Advanced Search<br>Database - MEDLINE Complete	Display
S24	AB "psychiatry* disease*" OR TI "psychiatry* disease*"	Search modes - Proximity	Interface - EBSCOhost Research Databases<br>Search Screen - Advanced Search<br>Database - MEDLINE Complete	Display

S23	AB "psychiatry* disorder*" OR TI "psychiatry* disorder*"	Search modes - Proximity	Interface - EBSCOhost Research Databases<br>Search Screen - Advanced Search<br>Database - MEDLINE Complete	Display
S22	AB "mental* health" OR TI "mental* health"	Search modes - Proximity	Interface - EBSCOhost Research Databases<br>Search Screen - Advanced Search<br>Database - MEDLINE Complete	Display
S21	AB "mental* impairment*" OR TI "mental* impairment*"	Search modes - Proximity	Interface - EBSCOhost Research Databases<br>Search Screen - Advanced Search<br>Database - MEDLINE Complete	Display
S20	AB "mental* issue*" OR TI "mental* issue*"	Search modes - Proximity	Interface - EBSCOhost Research Databases<br>Search Screen - Advanced Search<br>Database - MEDLINE Complete	Display
S19	AB "mental* distress" OR TI "mental* distress"	Search modes - Proximity	Interface - EBSCOhost Research Databases<br>Search Screen - Advanced Search<br>Database - MEDLINE Complete	Display
S18	AB "mental* crisis" OR TI "mental* crisis"	Search modes - Proximity	Interface - EBSCOhost Research Databases<br>Search Screen - Advanced Search<br>Database - MEDLINE Complete	Display
S17	AB "mental* problem*" OR TI "mental* problem*"	Search modes - Proximity	Interface - EBSCOhost Research Databases<br>Search Screen - Advanced Search<br>Database - MEDLINE Complete	Display

S16	AB "mental* illness*" OR TI "mental* illness*"	Search modes - Proximity	Interface - EBSCOhost Research Databases<br>Search Screen - Advanced Search<br>Database - MEDLINE Complete	Display
S15	AB "mental* ill" OR TI "mental* ill"	Search modes - Proximity	Interface - EBSCOhost Research Databases<br>Search Screen - Advanced Search<br>Database - MEDLINE Complete	Display
S14	AB "mental* disorder*" OR TI "mental* disorder*"	Search modes - Proximity	Interface - EBSCOhost Research Databases<br>Search Screen - Advanced Search<br>Database - MEDLINE Complete	Display
S13	AB "mental* disease*" OR TI "mental* disease*"	Search modes - Proximity	Interface - EBSCOhost Research Databases<br>Search Screen - Advanced Search<br>Database - MEDLINE Complete	Display
S12	AB "psychiatry* disease*" OR TI "psychiatry* disease*"	Search modes - Proximity	Interface - EBSCOhost Research Databases<br>Search Screen - Advanced Search<br>Database - MEDLINE Complete	Display
S11	AB "psychiatry* disorder*" OR TI "psychiatry* disorder*"	Search modes - Proximity	Interface - EBSCOhost Research Databases<br>Search Screen - Advanced Search<br>Database - MEDLINE Complete	Display
S10	AB "mental* health" OR TI "mental* health"	Search modes - Proximity	Interface - EBSCOhost Research Databases<br>Search Screen - Advanced Search<br>Database - MEDLINE Complete	Display

S9	AB "mental* impairment*" OR TI "mental* impairment*"	Search modes - Proximity	Interface - EBSCOhost Research Databases<br>Search Screen - Advanced Search<br>Database - MEDLINE Complete	Display
S8	AB "mental* issue*" OR TI "mental* issue*"	Search modes - Proximity	Interface - EBSCOhost Research Databases<br>Search Screen - Advanced Search<br>Database - MEDLINE Complete	Display
S7	AB "mental* distress" OR TI "mental* distress"	Search modes - Proximity	Interface - EBSCOhost Research Databases<br>Search Screen - Advanced Search<br>Database - MEDLINE Complete	Display
S6	AB "mental* crisis" OR TI "mental* crisis"	Search modes - Proximity	Interface - EBSCOhost Research Databases<br>Search Screen - Advanced Search<br>Database - MEDLINE Complete	Display
S5	AB "mental* problem*" OR TI "mental* problem*"	Search modes - Proximity	Interface - EBSCOhost Research Databases<br>Search Screen - Advanced Search<br>Database - MEDLINE Complete	Display
S4	AB "mental* illness*" OR TI "mental* illness*"	Search modes - Proximity	Interface - EBSCOhost Research Databases<br>Search Screen - Advanced Search<br>Database - MEDLINE Complete	Display
S3	AB "mental* ill" OR TI "mental* ill"	Search modes - Proximity	Interface - EBSCOhost Research Databases<br>Search Screen - Advanced Search<br>Database - MEDLINE Complete	Display

S2	AB "mental* disorder*" OR TI "mental* disorder*"	Search modes - Proximity	Interface - EBSCOhost Research Databases<br>Search Screen - Advanced Search<br>Database - MEDLINE Complete	Display
S1	AB "mental* disease*" OR TI "mental* disease*"	Search modes - Proximity	Interface - EBSCOhost Research Databases<br>Search Screen - Advanced Search<br>Database - MEDLINE Complete	Display
